# The comparison of epidemiological characteristics between confirmed and clinically diagnosed cases with COVID-19 during the early epidemic in Wuhan, China

**DOI:** 10.1186/s41256-021-00200-8

**Published:** 2021-05-28

**Authors:** Fang Shi, Haoyu Wen, Rui Liu, Jianjun Bai, Fang Wang, Sumaira Mubarik, Xiaoxue Liu, Yong Yu, Qiumian Hong, Jinhong Cao, Chuanhua Yu

**Affiliations:** 1 Department of Epidemiology and Biostatistics, School of Health Sciences, Wuhan University, 430071 Wuhan, China; 2 NHC Key lab of Radiation Biology, Jilin University, 130021 Changchun, China; 3 School of Public Health and Management, Hubei University of Medicine, 442000 Shiyan, China; 4 Department of Global Health, School of Health Sciences, Wuhan University, 430071 Wuhan, China; 5 Global Health Institute, Wuhan University, 430072 Wuhan, China

**Keywords:** COVID-19, Wuhan city, Epidemiology, Clinical diagnosis, Risk factor, Effective reproduction number

## Abstract

**Background:**

To put COVID-19 patients into hospital timely, the clinical diagnosis had been implemented in Wuhan in the early epidemic. Here we compared the epidemiological characteristics of laboratory-confirmed and clinically diagnosed cases with COVID-19 in Wuhan.

**Methods:**

Demographics, case severity and outcomes of 29,886 confirmed cases and 21,960 clinically diagnosed cases reported between December 2019 and February 24, 2020, were compared. The risk factors were estimated, and the effective reproduction number (Rt) of SARS-CoV-2 was also calculated.

**Results:**

The age and occupation distribution of confirmed cases and clinically diagnosed cases were consistent, and their sex ratio were 1.0 and 0.9, respectively. The epidemic curve of clinical diagnosis cases was similar to that of confirmed cases, and the city centers had more cumulative cases and higher incidence density than suburbs in both of two groups. The proportion of severe and critical cases (21.5 % vs. 14.0 %, *P* < 0.0001) and case fatality rates (5.2 % vs. 1.2 %, *P* < 0.0001) of confirmed cases were all higher than those of clinically diagnosed cases. Risk factors for death we observed in both of two groups were older age, male, severe or critical cases. Rt showed the same trend in two groups, it dropped below 1.0 on February 6 among confirmed cases, and February 8 among clinically diagnosed cases.

**Conclusions:**

The demographic characteristics and spatiotemporal distributions of confirmed and clinically diagnosed cases are roughly similar, but the disease severity and clinical outcome of clinically diagnosed cases are better than those of confirmed cases. In cases when detection kits are insufficient during the early epidemic, the implementation of clinical diagnosis is necessary and effective.

**Supplementary Information:**

The online version contains supplementary material available at 10.1186/s41256-021-00200-8.

## Introduction

In December 2019, a highly pathogenic coronavirus, severe acute respiratory syndrome coronavirus 2 (SARS-CoV-2), was recognized in Wuhan, China, and then sustained transmission has been seen throughout and outside China. The World Health Organization named the pneumonia caused by SARS-CoV-2 as Corona Virus Disease 2019 (COVID-19) [1], and announced that new coronary pneumonia has developed into a “pandemic” on 11 March 2020.

Massive measures have been taken by the government to curb the spread of COVID-19 in Wuhan, including the lockdown of Wuhan, which helped in limiting crowd movement to prevent infected cases from spreading to other areas [2–4]. The viral nucleic acid test (real-time reverse transcriptase–polymerase chain reaction [RT-PCR] assay or genome sequencing) is considered as the diagnostic gold standard of COVID-19 [5, 6]. Before February 8, 2020, only patients who had positive results on virus nucleic acid tests were regarded as laboratory-confirmed cases across China (To be consistent with the Chinese government’s reports, the following laboratory-confirmed cases are referred to as confirmed cases). However, due to a large number of patients, insufficient testing kits, and bottlenecks in laboratory testing capacity, the nucleic acid detection failed to meet clinical needs, and patients in Hubei Province could not be admitted to the hospital for treatment in time [7]. It is important to admit patients into hospitals as soon as possible since a deferred admission may turn patients critical and lead to more infections. To raise the hospital admission and improve the efficiency of treatment, the broadened diagnostic criteria were used and the designation ‘clinically diagnosed cases’ emerged. According to the revised fifth version of the guideline over the diagnosis and treatment of COVID-19 issued on Feb 8, 2020, jointly released by the National Health Commission of China and the State Administration of Traditional Chinese Medicine, clinical diagnosis was being used in Hubei Province only [5]. Without laboratory confirmation, clinically diagnosed cases were diagnosed by symptoms, exposures and CT scan only [5, 8]. Thanks to the revision in the diagnostic criteria, the patient admission rate has surged immediately. In the later period, the detection of COVID-19 had been greatly improved, the laboratory diagnostic ability could be met needs, and the suspected cases in Hubei Province could be rapidly detected. Therefore, the updated guideline known as the sixth edition issued on Feb 19, 2020, abolished different epidemic-related standards inside and outside Hubei Province, “clinically diagnosed cases” were no longer listed [6]. In addition, the number of clinically diagnosed cases was revised on Feb 24, 2020 [7].

To inform evidence-based decisions, more information relevant to the epidemiology of COVID-19 was urgently needed [9]. There are many studies on the confirmed cases [10–12], but no description of the epidemiology of clinically diagnosed cases has been seen. Here is a comparison and generalization of epidemiological characteristics of confirmed and clinically diagnosed cases with COVID-19 during the early epidemic in Wuhan.

## Methods

### Data sources

This was a retrospective study. All data from December 8, 2019 (date of the first onset) to 24 February 2020, were extracted from China’s Infectious Disease Information System. Details of data collection are provided elsewhere [13]. After excluding duplicate cases and those who were unable to obtain a unique identifying card, a total of 29,886 confirmed cases and 21,960 clinically diagnosed cases with COVID-19 in Wuhan were eligible for this study finally.

### Variables

COVID-19 was classified into mild type, moderate type, severe type as well as critical type according to disease severity, the detailed classification criteria were shown in Supplementary Table S1. The date of onset was defined as the day when the symptom was observed. The proportion of severe and critical cases was defined as (severe cases + critical cases) / (mild cases + moderate cases + severe cases + critical cases). Case fatality rates were calculated as the number of deaths divided by the total number of cases. Incidence density was estimated as the number of cases divided by the number of permanent resident population, which was collected from the Hubei Statistical Yearbook 2020.

### Case Definitions

According to the 5th edition of the guideline over the diagnosis and treatment of COVID-19 [5], confirmed cases were patients who had positive SARS-CoV-2 results after conducting RT-PCR assay or high-throughput sequencing of nasal and pharyngeal swab specimens. Clinically diagnosed cases were suspected cases with lung imaging features consistent with coronavirus pneumonia. Bilateral distribution of patchy shadows and ground-glass opacity were typical hallmarks of CT scan for COVID-19 [8].

### Statistical analysis

All data were recorded and sorted in Excel. Continuous variables were described using median and interquartile range (IQR) when the data did not obey normal distribution. Categorical variables were described by frequency, rate and percentage. The epidemic curve was built and maps of Wuhan at the county-level were drawn. Case severity and clinical outcomes between confirmed cases and clinically diagnosed cases were compared using Chi square tests or Kolmogorov-Smirnov *Z* tests. Univariable and multivariable logistic-regression analysis was performed to ascertain the risk factors for severity or death. The logistic-regression model did not include variable ‘days from onset to diagnosis’ because there was collinearity between variable ‘date of onset’ and ‘days from onset to diagnosis’. Odds ratio (OR) and its 95 % confidence intervals were calculated, corresponding forest-plot was drawn. The effective reproduction number (Rt), which is an indicator to measure the transmission of infectious diseases, is defined as the mean number of secondary cases generated by a typical primary case at time t in a population. When Rt is less than 1, the epidemic of infectious diseases will be gradually controlled; when Rt is greater than 1, infectious diseases will continue to spread, suggesting that prevention and control measures need to be optimized or strengthened. We applied the method developed by Anne Cori [14] to estimate Rt and its 95 % credible interval via a weekly sliding average. Referring to previous epidemiological surveys of Wuhan in the early stage of the COVID-19 outbreak, the parameters of serial interval distribution (gamma distribution, mean = 7.5 days, standard deviation = 3.4 days) were cited [15]. SPSS version 26.0 and R version 4.0 were used for statistical analyses and ArcGIS version 10.7 was used for cartography.

## Results

### Baseline epidemiological characteristics

**Fig. 1:**
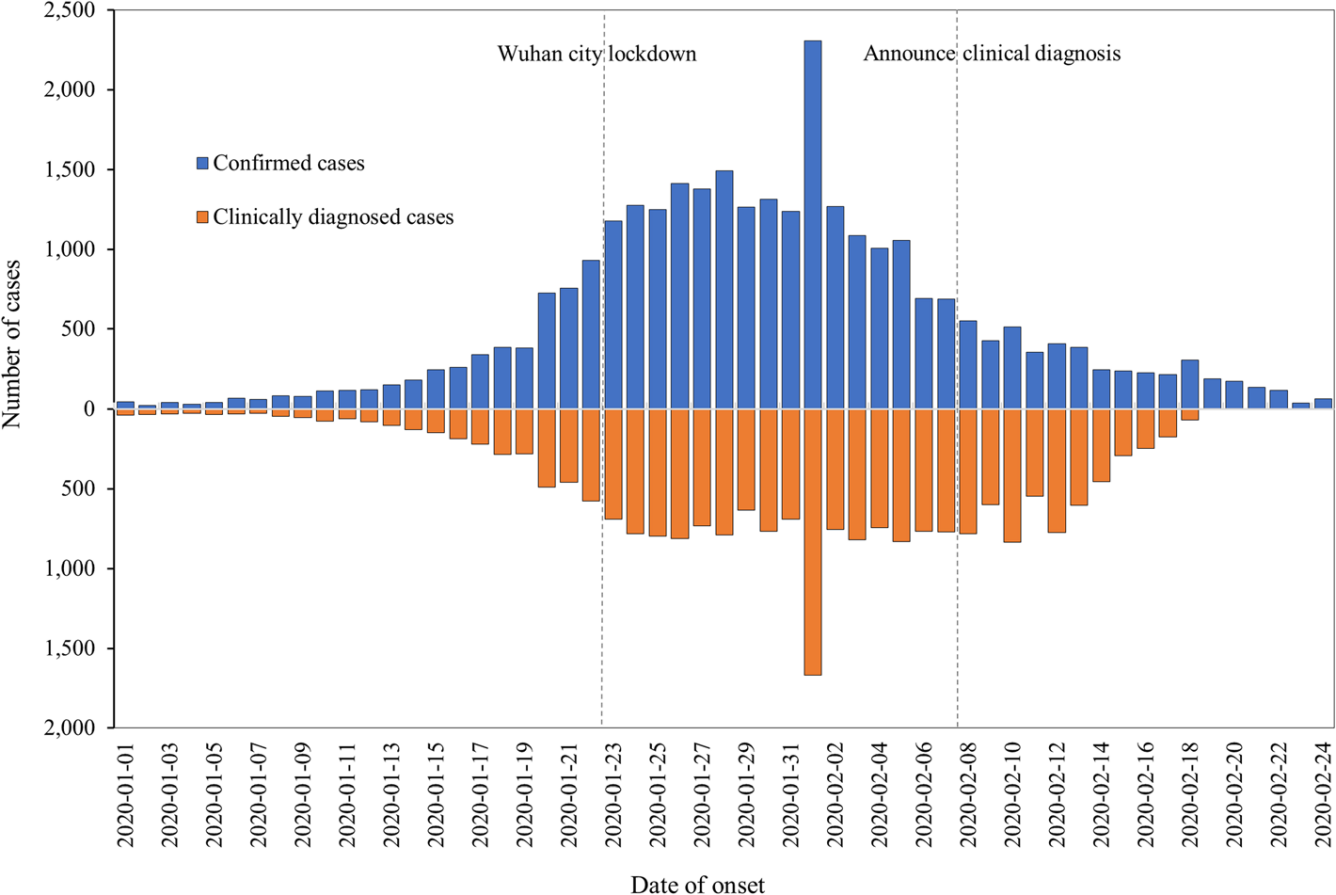
The epidemiological curves of confirmed cases and clinically diagnosed cases with COVID-19 in Wuhan from January 1 to February 24, 2020

**Fig. 2:**
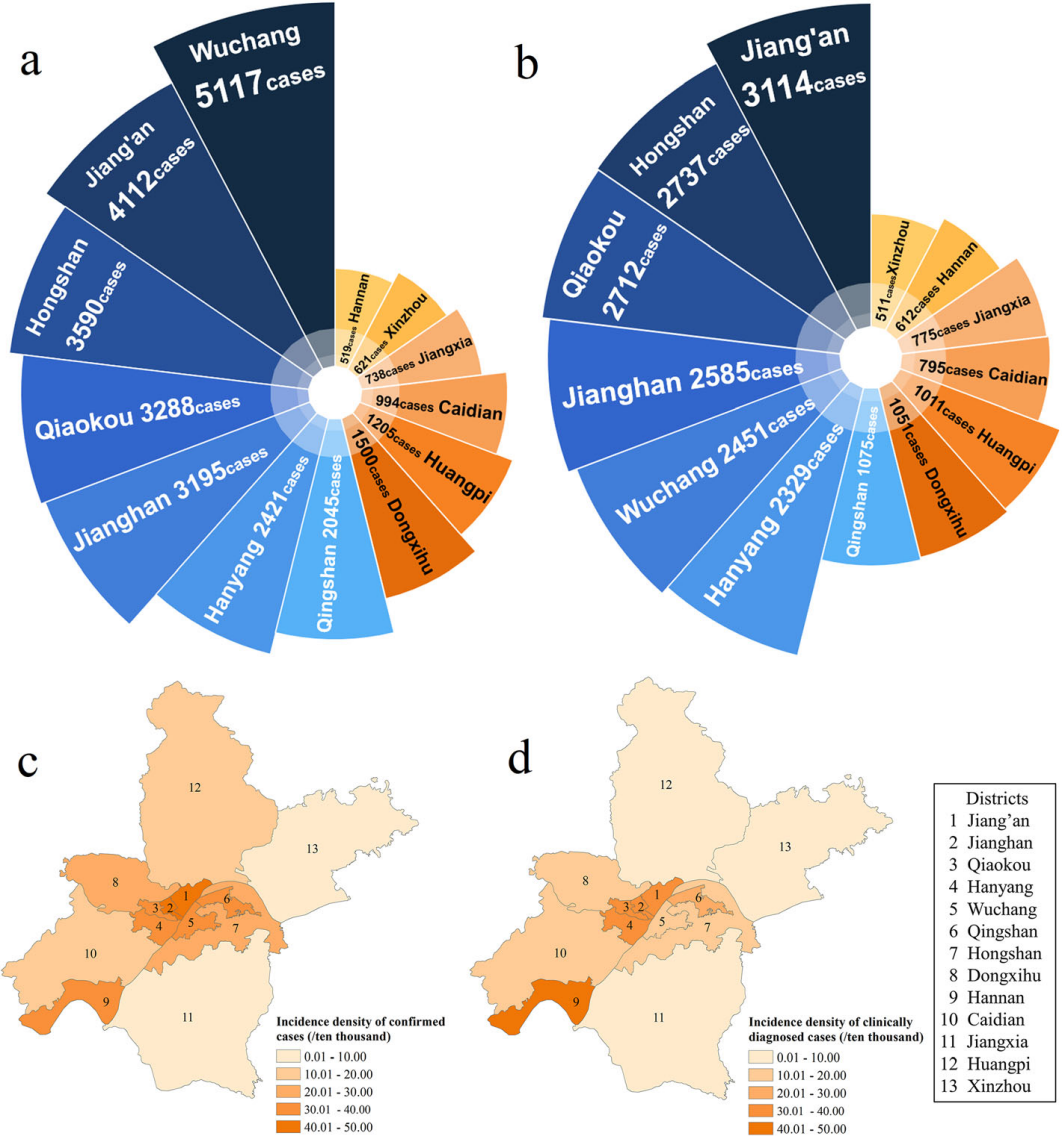
The cumulative numbers and incidence density of confirmed cases and clinically diagnosed cases with COVID-19 in 13 districts of Wuhan. (**a**) rose diagram of cumulative confirmed cases, (**b**) rose diagram of cumulative clinically diagnosed cases. The 7 districts with blue series belong to the city centers, and the 6 districts with orange series belong to the suburbs in Wuhan. (**c**) map of incidence density of confirmed cases, (**d**) map of incidence density of clinically diagnosed cases

As of 24 February 2020, a total of 29,886 confirmed cases and 21,960 clinically diagnosed cases with COVID-19 were included in this study. The epidemiological curves of clinically diagnosed cases were similar to that of confirmed cases, the peak of COVID-19 onset occurred between the Wuhan lockdown and February 8 (Fig. 1). The baseline characteristics of cases were shown in Table 1. The sex ratio of confirmed cases and clinically diagnosed cases were 1.0 and 0.9, and the age distribution and occupation distribution of the two groups were similar (Supplementary Fig. S1). The median intervals between onset and diagnosis of confirmed cases and clinically diagnosed cases were 9.0 (5.0–13.0) and 11.0 (5.0–18.0) days, respectively. As time goes on, the interval between onset and diagnosis had decreased significantly (Supplementary Fig. S2). The city centers have more cumulative cases and higher incidence density than suburbs in both of the two groups (Fig. 2 and Supplementary Table S2).

**Table 1:** Baseline Epidemiological Characteristics of Confirmed Cases and Clinically Diagnosed Cases in Wuhan

**Baseline Characteristics**	**No. Confirmed Cases (%)**	**No. Clinically Diagnosed Cases (%)**
Total	29886	21960
Age, median (IQR ^a^), years	57.0(44.0–67.0)	54.0(41.0–65.0)
Age group, years		
0~	186(0.6)	220(1.0)
10~	226(0.8)	173(0.8)
20~	1371(4.6)	1412(6.4)
30~	3720(12.4)	3280(14.9)
40~	4435(14.8)	3736(17.0)
50~	6347(21.2)	4851(22.1)
60~	7678(25.7)	5007(22.8)
70~	3979(13.3)	2285(10.4)
80~	1671(5.6)	839(3.8)
≥90	217(0.7)	121(0.6)
Missing	56(0.2)	36(0.2)
Sex		
Male	15059(50.4)	10165(46.3)
Female	14827(49.6)	11795(53.7)
Occupation		
Child and student	439(1.5)	482(2.2)
Cadre	1489(5.0)	1119(5.1)
Freelancer	203(0.7)	194(0.9)
Physical labor	712(2.4)	641(2.9)
Public service staff	1816(6.1)	1477(6.7)
Housework or unemployed	5684(19.0)	5401(24.6)
Retirees	10012(33.5)	6114(27.8)
Farmer or pastoral worker	1390(4.7)	1002(4.6)
Healthcare worker	1188(4.0)	1211(5.5)
Missing	6953(23.3)	4319(19.7)
Case severity		
Mild	18192(60.9)	11326(51.6)
Moderate	4148(13.9)	7446(33.9)
Severe	5278(17.7)	2749(12.5)
Critical	823(2.8)	315(1.4)
Missing	1445(4.8)	124(0.6)
Death or not		
Not	28322(94.8)	21703(98.8)
Yes	1564(5.2)	257(1.2)
Date of onset		
Before Dec 31, 2019	135(0.5)	92(0.4)
Jan 1–10, 2020	591(2.0)	388(1.8)
Jan 11–20, 2020	2921(9.8)	1981(9.0)
Jan 21–31, 2020	13502(45.2)	7741(35.3)
Feb 1–10, 2020	9600(32.1)	8583(39.1)
Feb 11–20, 2020	2758(9.2)	3172(14.4)
Feb 21–24, 2020	355(1.2)	——
Missing	24(0.1)	3(0.0)
Days from onset to diagnosis, median (IQR)	9.0 (5.0–13.0)	11.0 (5.0–18.0)
District of residence		
City centre	23768(79.5)	17003(77.4)
Suburb	5577(18.7)	4755(21.7)
Outside Wuhan	339(1.1)	173(0.8)
Missing	202(0.7)	29(0.1)
Level of hospital		
Tertiary hospital	21622(72.3)	18598(84.7)
Primary and secondary hospital	8166(27.3)	3362(15.3)
Missing	98(0.3)	——

^a^*IQR* interquartile range

### Severity of illness

The proportion of severe and critical types in confirmed cases was higher than that in clinically diagnosed cases (21.5 % vs. 14.0 %, *P* < 0.0001). As given in Supplementary Table S3, the epidemiological characteristics of COVID-19 varied by the classification of severity. The median ages of the severe and critical cases were higher than mild and moderate cases, and the proportion of severe and critical cases increased with age (Supplementary Fig. S3). The proportion of severe and critical cases in males was higher than that in females (53.6 % vs. 43.7 % in confirmed cases, and 60.6 % vs. 36.4 % in clinically diagnosed cases), and a later date of onset was associated with the milder disease. The proportion of severe and critical cases in confirmed and clinically diagnosed cases all decreased over time (Supplementary Fig. S4). Univariable and multivariable logistic-regression model showed that age greater than 60 years, males, special occupations (such as housework or unemployed, retirees, and healthcare worker) and earlier date of onset were risk factors for severity in both confirmed cases and clinically diagnosed cases (Supplementary Table S4 and Fig. S5).

### Analysis of Deaths

**Fig. 3:**
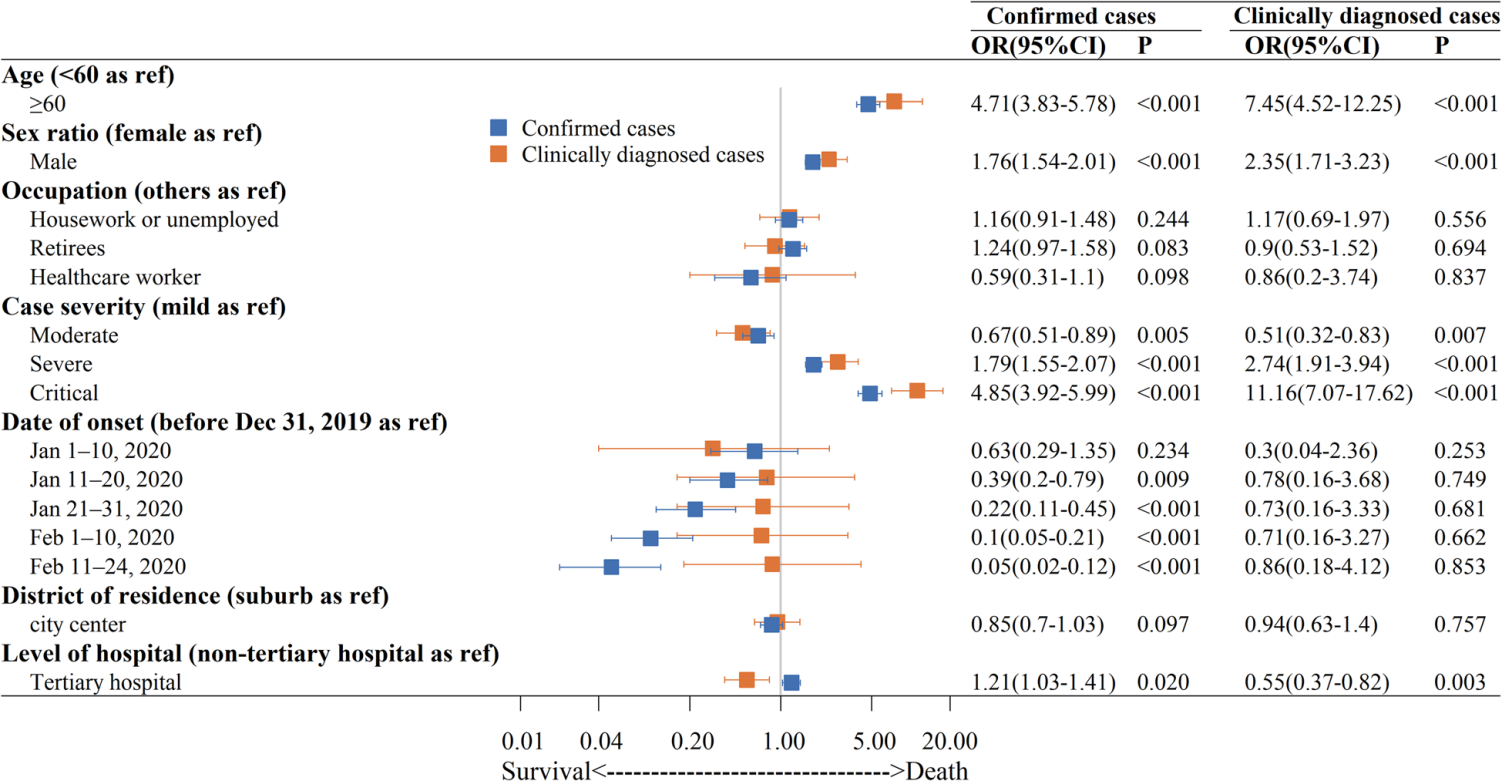
Risk factors for death in COVID-19 patients from multivariable logistic-regression analysis

The case fatality rates of confirmed cases and clinically diagnosed cases with COVID*-*19 were 5.2 and 1.5 %, respectively (Table 2). The median age and sex ratio of deaths were significantly higher than those who did not die both in confirmed cases and clinically diagnosed cases. The case fatality rates of severe and critical cases were higher than those of mild and moderate cases, respectively. The deaths of confirmed cases were concentrated in city centers, while the deaths of clinically diagnosed cases were mainly concentrated in the suburbs (Supplementary Fig. S6). The percentage of deaths decreased over time during the early epidemic (Supplementary Fig. S4). Univariable and multivariable logistic-regression was developed to predict the risk factors for death from COVID-19. Age greater than 60 years, males, and more serious case severity were found to be related to an increased risk of death in both of two groups (Fig. 3 and Supplementary Table S5).

**Table 2:** Epidemiological Characteristics of Death Cases and Cases not Dead in Wuhan

Characteristics	Confirmed Cases			Clinically Diagnosed Cases			***P***
	Death (%)	Not Dead (%)	Case Fatality Rate (%)	Death (%)	Not Dead (%)	Case Fatality Rate (%)	
Total	1564	28322	5.2	257	21703	1.2	<0.001
Age, median (IQR ^a^), years	70.0(63.0–79.0)	56.0(53.0–66.0)	——	70.0(62.0–78.0)	54.0(40.0–65.0)	——	——
Age group, years							
0~	0(0.0)	186(0.7)	0.0	0(0.0)	220(1.0)	0.0	——
10~	1(0.1)	225(0.8)	0.4	1(0.4)	172(0.8)	0.6	1.000
20~	6(0.4)	1365(4.8)	0.4	1(0.4)	1411(6.5)	0.1	0.120
30~	20(1.3)	3700(13.1)	0.5	3(1.2)	3277(15.1)	0.1	0.001
40~	49(3.1)	4386(15.5)	1.1	9(3.5)	3727(17.2)	0.2	<0.001
50~	175(11.2)	6172(21.8)	2.8	34(13.2)	4817(22.2)	0.7	<0.001
60~	455(29.1)	7223(25.5)	5.9	77(30.0)	4930(22.7)	1.5	<0.001
70~	473(30.2)	3506(12.4)	11.9	80(31.1)	2205(10.2)	3.5	<0.001
80~	300(19.2)	1371(4.8)	18.0	45(17.5)	794(3.7)	5.4	<0.001
≥90	52(3.3)	165(0.6)	24.0	6(2.3)	115(0.5)	5.0	<0.001
Missing	33(2.1)	23(0.1)	58.9	1(0.4)	35(0.2)	2.8	<0.001
Sex							
Male	1026(65.6)	14033(49.5)	6.8	177(68.9)	9988(46.0)	1.7	<0.001
Female	538(34.4)	14289(50.5)	3.6	80(31.1)	11715(54.0)	0.7	<0.001
Occupation							
Child and student	2(0.1)	437(1.5)	0.5	0(0.0)	482(2.2)	0.0	0.227
Cadre	31(2.0)	1458(5.1)	2.1	4(1.6)	1115(5.1)	0.4	<0.001
Freelancer	6(0.4)	197(0.7)	3.0	1(0.4)	193(0.9)	0.5	0.143
Physical labor	19(1.2)	693(2.4)	2.7	4(1.6)	637(2.9)	0.6	0.004
Public service staff	23(1.5)	1793(6.3)	1.3	2(0.8)	1475(6.8)	0.1	<0.001
Housework or unemployed	267(17.1)	5417(19.1)	4.7	57(22.2)	5344(24.6)	1.1	<0.001
Retirees	753(48.1)	9259(32.7)	7.5	104(40.5)	6010(27.7)	1.7	<0.001
Farmer or pastoral worker	51(3.3)	1339(4.7)	3.7	15(5.8)	987(4.5)	1.5	0.001
Healthcare worker	12(0.8)	1176(4.2)	1.0	2(0.8)	1209(5.6)	0.2	0.007
Missing	400(25.6)	6553(23.1)	5.8	68(26.5)	4251(19.6)	1.6	<0.001
Case severity							
Mild	603(38.6)	17589(62.1)	3.3	79(30.7)	11247(51.8)	0.7	<0.001
Moderate	69(4.4)	4079(14.4)	1.7	31(12.1)	7415(34.2)	0.4	<0.001
Severe	477(30.5)	4801(17.0)	9.0	73(28.4)	2676(12.3)	2.7	<0.001
Critical	210(13.4)	613(2.2)	25.5	39(15.2)	276(1.3)	12.4	<0.001
Missing	205(13.1)	1240(4.4)	14.2	35(13.6)	89(0.4)	28.2	<0.001
Date of onset							
Before Dec 31, 2019	29(1.9)	106(0.4)	21.5	4(1.6)	88(0.4)	4.3	<0.001
Jan 1–10, 2020	132(8.4)	459(1.6)	22.3	9(3.5)	379(1.7)	2.3	<0.001
Jan 11–20, 2020	392(25.1)	2529(8.9)	13.4	30(11.7)	1951(9.0)	1.5	<0.001
Jan 21–31, 2020	769(49.2)	12733(45.0)	5.7	95(37.0)	7646(35.2)	1.2	<0.001
Feb 1–10, 2020	215(13.7)	9385(33.1)	2.2	84(32.7)	8499(39.2)	1.0	<0.001
Feb 11–20, 2020	26(1.7)	2732(9.6)	0.9	32(12.5)	3140(14.5)	1.0	0.796
Feb 21–24, 2020	0(0.0)	355(1.3)	0.0	0(0.0)	0(0.0)	——	——
Missing	1(0.1)	23(0.1)	4.2	3(1.2)	——	0.0	——
Days from onset to diagnosis, median (IQR)	10.0(7.0–14.0)	9.0(4.0–13.0)	——	8.0(3.0–15.0)	11.0(5.0–18.0)	——	——
District of residence							
City centre	1208(77.2)	22560(79.6)	5.1	169(65.8)	16834(77.6)	1.0	<0.001
Suburb	227(14.5)	5350(18.9)	4.1	58(22.6)	4697(21.6)	1.2	<0.001
Outside Wuhan	33(2.1)	306(1.1)	9.7	1(0.4)	172(0.8)	0.6	<0.001
Missing	96(6.1)	106(0.4)	47.5	29(11.3)	——	——	——
Level of hospital							
Tertiary hospital	1254(80.2)	20368(71.9)	5.8	205(79.8)	18393(84.7)	1.1	<0.001
Primary / secondary hospital	310(19.8)	7856(27.8)	3.8	52(20.2)	3310(15.3)	1.5	<0.001
Missing	——	98(0.3)	——	——	——	——	——

^a^*IQR* interquartile range

### Rt of confirmed and clinically diagnosed cases

**Fig. 4:**
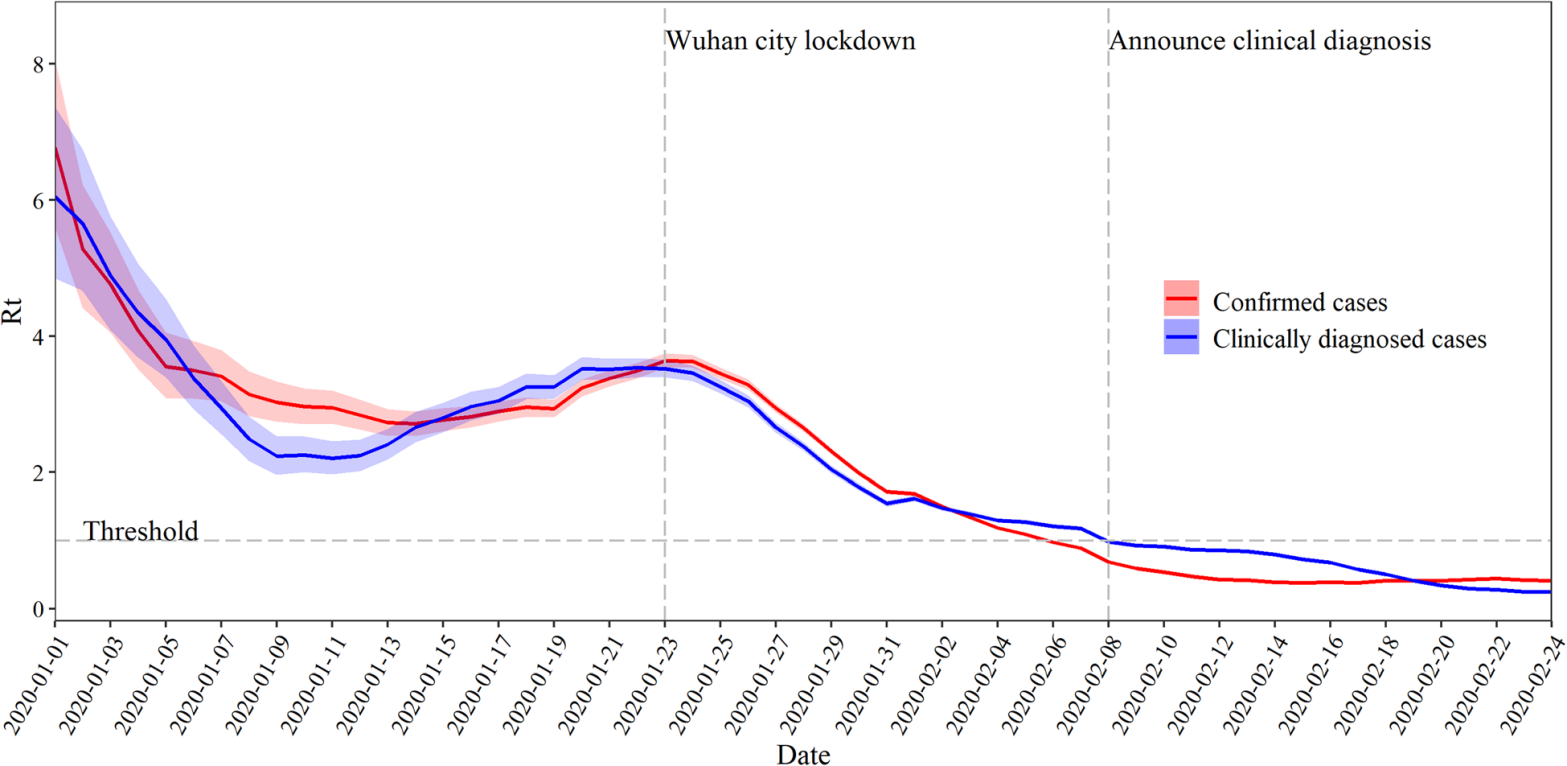
Estimated Rt of confirmed cases and clinically diagnosed cases with COVID-19 in Wuhan, China. The 95 % confidence intervals are presented as red or blue shading. The gray horizontal line indicates Rt = 1, below which suggests that the outbreak is gradually controlled

Rt curves showed the same trend in the two groups. For confirmed (or clinically diagnosed) cases, Rt fluctuated above 2.0 before January 30, reached a peak of 3.64 (3.54) on January 23 (January 22), and further declined after Wuhan city lockdown, finally decreased to below 1.0 after February 6 (February 8). The trend of Rt was shown in Fig. 4.

## Discussion

Wuhan bore the brunt during the epidemic. To put patients into hospital and under treatment timely, the clinically diagnosed cases had been identified from February 8 to February 18. Besides, the number of clinically diagnosed cases was revised on February 24 [7]. The study is a comparison of the 29,886 confirmed cases and 21,960 clinically diagnosed cases with COVID-19 in the early stage of the epidemic in Wuhan. To the best of our knowledge, no other papers discussed these two types of patients and how similar or dissimilar they are in describing the epidemic.

This study showed that the demographic characteristics of confirmed and clinically diagnosed cases were similar, suggesting that clinical diagnosis was effective which could accurately detect the vast majority of COVID-19 patients. The age and occupational distribution of clinically diagnosed cases were coincident with those of confirmed cases. This study showed that people of all ages were susceptible to the virus, but most patients were middle-aged and old people. Patients aged over 60 years accounted for 41·0 % of confirmed cases and 36·6 % of clinically diagnosed cases. Age-related decline and dysregulation of immune function give rise to the heightened vulnerability to COVID-19 in the elderly [16]. Retirees accounted for the largest proportion of patient’s occupations, which may be due to the fact that retirees are usually older adults. The median (IQR) interval between onset and diagnosis in confirmed cases was 9 (5–13) days, which were slightly shorter than that in clinically diagnosed cases [11 (5–17) days]. Patients with early-onset received the nucleic acid diagnosis preferentially, while the patients with late-onset could not receive RT-PCR or genome sequencing of SARS-COV-2 in time when the detection kits were insufficient. Besides, Wuhan experienced the peak of the COVID-19 outbreak between the Wuhan lockdown and February 8, which accelerated the consumption of detection reagents and the backlog of patients. At this time, it was necessary to carry out a clinical diagnosis for patients who had already developed symptoms but could not be confirmed by the laboratory, since the condition will worsen if they could not be isolated or admitted promptly. The interval between onset and diagnosis had seen a continuous decrease as time went by, meaning the implementation of clinical diagnosis effectively shortened the duration before diagnosis in the early stage of the epidemic. When the median interval between onset and diagnosis was shortened to 2 days, the clinical diagnosis was canceled on February 19, 2020.

There are 13 districts in Wuhan: Jiang’an, Jianghan, Qiaokou, Hanyang, Wuchang, Qingshan, Hongshan, Dongxihu, Hannan, Caidian, Jiangxia, Huangpi and Xinzhou, the first seven of which are city centers. The cumulative number of patients in the city centers was 4.7 and 3.6 times those in the suburbs among confirmed cases and clinically diagnosed cases, respectively. The city centers have a large number of permanent residents and floating populations, which is prone to the spread of the virus. Besides, the city centers have abundant and concentrated medical resources, for example, there are more tertiary hospitals (Supplementary Fig. S7), which makes it easier for the infected people there to be diagnosed than suburbanites. The geographical distribution of incidence density in confirmed and clinically diagnosed cases was similar, the incidence density in city centers was 2.8 times that in suburbs among confirmed cases, and 2.4 times among clinically diagnosed cases. City centers were hardest-hit regions of the COVID-19 epidemic, it is necessary to carry out key monitoring, prevention and control of the epidemic in city centers.

The proportion of severe and critical types in confirmed cases was significantly higher than that in clinically diagnosed cases (21.5 % vs. 14.0 %, *P* < 0.05). Therefore, the case fatality rate of confirmed cases was considerably significantly above that of clinically diagnosed patients (5.2 % vs. 1.5 %, *P* < 0.05). In the case of limited detection reagents, severe and critical cases received the nucleic acid diagnosis preferentially. Approximately 67 % of severe and critical patients were laboratory-confirmed, while only 54 % of mild and moderate patients obtained the virus nucleic acid tests. Some critical patients who progressed to acute respiratory distress syndrome (ARDS) after mild symptoms for 7–8 days had been observed [17], implying the early recognition of infected cases is extremely important and mild patients should also receive early treatment to avoid becoming critically ill [15]. Therefore, it was necessary to carry out clinical diagnosis under the condition of a large backlog of suspected cases in Wuhan in the early stage of the COVID-19 epidemic. Besides, the geographical distributions of dead confirmed cases and dead clinically diagnosed cases were diverse. The dead confirmed cases were concentrated in city centers, while the deaths of clinically diagnosed cases were mainly concentrated in the suburbs. Suburban residents might not get laboratory-confirmation promptly due to relatively deficient health resources here. For example, there are less than 5 tertiary-A hospitals in suburbs, but more than 20 tertiary-A hospitals in city centers. In regions with insufficient medical resources, clinical diagnosis is an important supplement to laboratory-diagnostic methods. Many COVID-19 patients who lived in the suburbs had benefited from clinical diagnosis and received timely treatment. Thanks to the health public measures including the implementation of clinical diagnosis, the proportion of severe and critical cases as well as case-fatality rate had a continuous decrease, meaning those measures is helpful to control the growth of severe and critical cases and death.

The common risk factors for severity and death of the two groups were evaluated. This study found that aging was a prominent risk factor for severe disease and death from COVID-19, which was consistent with early reports [11, 13, 18]. The immune function and organ reserve capacity of the elderly are receded, and they tend to have serious underlying illnesses, the older the age is, the more severe the disease is [19]. Infectious disease, especially acute infection will bring adverse prognosis and death risk to the elderly. The elderly should be regarded as the key population for epidemic prevention and control [20, 21]. Sex was also closely related to the severity and death. Research from Johns Hopkins University found that the average case fatality rate of males across 38 countries was 1.7 times higher than that of females [22]. In our study, case fatality rate of males was 1.9 times higher than that of females among confirmed cases, and 2.4 times among clinically diagnosed cases. The male bias in severity and mortality of COVID-19 stems from the pathogenesis of SARS-CoV-2 infection. X chromosome and estrogen protect females from lethal infection [21, 23]; besides, numerous studies indicted ACE2, which used by SARS-CoV-2 to enter into the host cells [24, 25], generally has a higher expression in males than in females; moreover, females and males vary in their susceptibility and response to viral infections, the number and activity of innate immune cells, and immune responses are higher in females than in males [26]. There were some influencing factors that had opposite effects on the clinical outcomes in the two groups. Later date of onset was associated with a better chance of survival for confirmed cases, with no association found for clinically diagnosed cases. Besides, the tertiary hospitals with better medical level were associated with better clinical outcomes in confirmed cases, but were associated with worse outcomes in clinically diagnosed cases. We speculated that was because mild clinically diagnosed cases often went to primary hospitals, while clinically diagnosed cases with more serious illnesses were diagnosed and treated in big hospitals. The reasonable shunt of clinically diagnosed cases eased the medical pressure of tertiary-A hospitals.

The transmission dynamics of COVID-19 were identical in confirmed and clinically diagnosed cases. Their Rt both declined rapidly from the peak after the lockdown of Wuhan, and further decreased to below 1 after clinical diagnosis. It proves that rapid public health responses including the Wuhan lockdown and the implementation of clinical diagnosis, have successfully contained the spread of SARS-CoV-2 and mitigated the development of the epidemic.

Our study has several limitations. Firstly, there were a few missing values that might slightly affect the result. Secondly, the clinical outcomes of COVID-19 cases in our study were followed up to February 24, 2020, when many patients had not been discharged, so the ultimate case fatality rate could not be calculated [27]. According to the National Health Commission of China, a total of 50,333 cases were confirmed with COVID-19 in Wuhan and 3869 died as of April 30, 2020, the case fatality rate was 7.7 %. It speculates that many patients died later. Thirdly, data reliability of the interval between onset and diagnosis depended on the patients, which might cause some recall bias. Finally, we once again reiterated that the results were based on the data of Wuhan where was the worst-hit region in China, so it should be prudent to extrapolate those data to areas with less epidemic.

## Conclusion

In summary, the demographic characteristics and spatiotemporal distributions of confirmed and clinically diagnosed cases were roughly similar, but the disease severity as well as clinical outcome of clinically diagnosed cases were better than those of confirmed cases. The proportion of severe and critical cases, case-fatality rate as well as Rt of the two groups both decreased over time, suggesting that the swift measures China took, including the Wuhan lockdown and the implementation of clinical diagnosis, have successfully mitigated the development of the epidemic. In cases when medical resources are insufficient to cover the viral nucleic acid test of all COVID-19 cases, clinical diagnosis is effective and necessary. Clinical diagnosis is helpful to shorten the interval between onset and diagnosis, quarantine or treat patients as soon as possible, and improve the cure rate.

## Supplementary information

Additional file 1.

## Data Availability

The data that support the findings of this study are available from the National Health Commission of the People’s Republic of China, but restrictions apply to the availability of these data, which were used under license for the current study, and so are not publicly available. Data are however available from the authors upon reasonable request and with permission of the National Health Commission of the People’s Republic of China.

## Abbreviations

SARS-CoV-2: Severe acute respiratory syndrome coronavirus 2
COVID-19: Corona Virus Disease 2019
RT-PCR: Real-time reverse transcriptase–polymerase chain reaction
IQR: Interquartile range
OR: Odds ratio
Rt: Effective reproduction number
