# Supplementary material for: The comparison of epidemiological characteristics between confirmed and clinically diagnosed cases with COVID-19 during the early epidemic in Wuhan, China
Source: Glob Health Res Policy. 2021 May 28;6:18. doi: 10.1186/s41256-021-00200-8 (PMC8161348; doi:10.1186/s41256-021-00200-8)
Supplement: Supplementary file 1 — Additional file 1. [file 41256_2021_200_MOESM1_ESM.docx]

**Supplementary Material**

**Table S1.** The Classification Criteria of Severity of COVID-19

| **Classification** | **Criteria** |
| --- | --- |
| Mild type | The clinical symptoms were mild, and no pneumonia was found on the chest computed tomography (CT) |
| Common type | Patient had fever, respiratory symptoms, and imaging manifestations of pneumonia |
| Severe type | Fulfilling one of the following three conditions: 1. Respiratory distress, respiratory rate ≥ 30 times/min; 2. In resting state, oxygen saturation ≤ 93%; 3. Partial arterial oxygen pressure (PaO2)/oxygen absorption concentration (FiO2) ≤ 300 mmHg (1 mmHg = 0.133 kPa). |
| Critical type | Fulfilling one of the following three conditions: 1. Patient had respiratory failure and needed mechanical ventilation; 2. Patient suffered from shock; 3. Patient had other organ failure and needed ICU monitoring. |

**Table S2.** Cumulative Number and Incidence Density of Confirmed and Clinically Diagnosed Cases in All Districts of Wuhan

| **District** | **Permanent Residents (Ten Thousand)** | **Cumulative Confirmed Cases** | **Incidence Density of Confirmed Cases (/Ten Thousand)** | **Cumulative Clinically Diagnosed Cases** | **Incidence Density of Clinically Diagnosed Cases (/Ten Thousand)** |
| --- | --- | --- | --- | --- | --- |
| Jiang'an | 96.28 | 4112 | 42.71 | 3114 | 32.34 |
| Jianghan | 72.98 | 3195 | 43.78 | 2585 | 35.42 |
| Qiaokou | 86.89 | 3288 | 37.84 | 2712 | 31.21 |
| Hanyang | 67.00 | 2421 | 36.13 | 2329 | 34.76 |
| Wuchang | 128.54 | 5117 | 39.81 | 2451 | 19.07 |
| Qingshan | 52.90 | 2045 | 38.66 | 1075 | 20.32 |
| Hongshan | 171.29 | 3590 | 20.96 | 2737 | 15.98 |
| Dongxihu | 60.13 | 1500 | 24.95 | 1051 | 17.48 |
| Hannan | 13.60 | 519 | 38.16 | 612 | 45.00 |
| Caidian | 78.49 | 994 | 12.66 | 795 | 10.13 |
| Jiangxia | 98.70 | 738 | 7.48 | 775 | 7.85 |
| Huangpi | 102.80 | 1205 | 11.72 | 1011 | 9.83 |
| Xinzhou | 91.60 | 621 | 6.78 | 511 | 5.58 |
| City centres | 675.88 | 23768 | 35.17 | 17003 | 25.16 |
| Suburbs | 445.32 | 5577 | 12.52 | 4755 | 10.68 |
| Wuhan | 1121.20 | 29345 | 26.17 | 21758 | 19.41 |

**Table S3.** Epidemiological Characteristics of Clinically Diagnosed Cases with Different Severities

| **Characteristics** | **Confirmed Cases** | | | | | **Clinically Diagnosed Cases** | | | | | ***P*** |
| --- | --- | --- | --- | --- | --- | --- | --- | --- | --- | --- | --- |
|  | **Mild (%)** | **Moderate (%)** | **Severe (%)** | **Critical (%)** | **Proportion of severe and critical cases (%)** | **Mild (%)** | **Moderate (%)** | **Severe (%)** | **Critical (%)** | **Proportion of severe and critical cases (%)** |  |
| **Total** | 18192 | 4148 | 5278 | 823 | 21.5 | 11326 | 7446 | 2749 | 315 | 14.0 | <0.001 |
| **Age, median (IQR ^a^), years** | 56.0(42.0–65.0) | 56.0(44.0–67.0) | 64.0(52.0–71.0) | 67.0(58.0–75.0) |  | 53.0(39.0–63.0) | 53.0(39.0–64.0) | 63.0(50.0–71.0) | 66.0(60.0–74.0) | —— | —— |
| **Age group, years** |  |  |  |  |  |  |  |  |  |  |  |
| 0~ | 132(0.7) | 40(1.0) | 2(0.0) | 1(0.1) | 1.7 | 110(1.0) | 93(1.2) | 16(0.6) | 1(0.3) | 7.7 | <0.001 |
| 10~ | 162(0.9) | 35(0.8) | 12(0.2) | 2(0.2) | 6.6 | 91(0.8) | 73(1.0) | 9(0.3) | 0(0.0) | 5.2 | 0.226 |
| 20~ | 936(5.1) | 225(5.4) | 117(2.2) | 12(1.5) | 10.0 | 748(6.6) | 558(7.5) | 95(3.5) | 3(1.0) | 7.0 | 0.005 |
| 30~ | 2579(14.2) | 490(11.8) | 423(8.0) | 22(2.7) | 12.7 | 1895(16.7) | 1136(15.3) | 237(8.6) | 1(0.3) | 7.3 | <0.001 |
| 40~ | 2994(16.5) | 657(15.8) | 552(10.5) | 53(6.4) | 14.2 | 2061(18.2) | 1348(18.1) | 278(10.1) | 20(6.3) | 8.0 | <0.001 |
| 50~ | 4055(22.3) | 921(22.2) | 937(17.8) | 134(16.3) | 17.7 | 2626(23.2) | 1625(21.8) | 522(19.0) | 52(16.5) | 11.9 | <0.001 |
| 60~ | 4518(24.8) | 1003(24.2) | 1605(30.4) | 246(29.9) | 25.1 | 2471(21.8) | 1612(21.6) | 788(28.7) | 113(35.9) | 18.1 | <0.001 |
| 70~ | 1994(11.0) | 512(12.3) | 1037(19.6) | 221(26.9) | 33.4 | 979(8.6) | 706(9.5) | 505(18.4) | 81(25.7) | 25.8 | <0.001 |
| 80~ | 728(4.0) | 226(5.4) | 507(9.6) | 112(13.6) | 39.4 | 308(2.7) | 239(3.2) | 249(9.1) | 33(10.5) | 34.0 | 0.010 |
| ≥90 | 71(0.4) | 36(0.9) | 79(1.5) | 16(1.9) | 47.0 | 24(0.2) | 40(0.5) | 45(1.6) | 10(3.2) | 46.2 | 0.888 |
| Missing | 23(0.1) | 3(0.1) | 7(0.1) | 4(0.5) | 29.7 | 13(0.1) | 16(0.2) | 5(0.2) | 1(0.3) | 17.1 | 0.209 |
| **Sex** |  |  |  |  |  |  |  |  |  |  |  |
| Male | 8990(49.4) | 2094(50.5) | 2733(51.8) | 463(56.3) | 22.4 | 5168(45.6) | 3422(46.0) | 1324(48.2) | 191(60.6) | 15.0 | <0.001 |
| Female | 9202(50.6) | 2054(49.5) | 2545(48.2) | 360(43.7) | 20.5 | 6158(54.4) | 4024(54.0) | 1425(51.8) | 124(39.4) | 13.2 | <0.001 |
| **Occupation** |  |  |  |  |  |  |  |  |  |  |  |
| Child and student | 321(1.8) | 77(1.9) | 14(0.3) | 1(0.1) | 3.6 | 259(2.3) | 197(2.6) | 24(0.9) | 1(0.3) | 5.2 | 0.259 |
| Cadre | 1005(5.5) | 217(5.2) | 203(3.8) | 16(1.9) | 15.2 | 526(4.6) | 501(6.7) | 81(2.9) | 4(1.3) | 7.6 | <0.001 |
| Freelancer | 115(0.6) | 45(1.1) | 27(0.5) | 6(0.7) | 17.1 | 56(0.5) | 108(1.5) | 24(0.9) | 5(1.6) | 15.0 | 0.579 |
| Physical labor | 484(2.7) | 112(2.7) | 86(1.6) | 14(1.7) | 14.4 | 381(3.4) | 199(2.7) | 53(1.9) | 7(2.2) | 9.4 | 0.005 |
| Public service staff | 1393(7.7) | 192(4.6) | 163(3.1) | 10(1.2) | 9.8 | 937(8.3) | 407(5.5) | 103(3.7) | 4(1.3) | 7.4 | 0.014 |
| Housework or unemployed | 3290(18.1) | 861(20.8) | 1133(21.5) | 241(29.3) | 24.9 | 2576(22.7) | 2061(27.7) | 669(24.3) | 91(28.9) | 14.1 | <0.001 |
| Retirees | 5563(30.6) | 1459(35.2) | 2396(45.4) | 350(42.5) | 28.1 | 2909(25.7) | 1919(25.8) | 1108(40.3) | 139(44.1) | 20.5 | <0.001 |
| Farmer or pastoral worker | 883(4.9) | 261(6.3) | 164(3.1) | 39(4.7) | 15.1 | 664(5.9) | 208(2.8) | 109(4.0) | 21(6.7) | 13.0 | 0.150 |
| Healthcare worker | 803(4.4) | 125(3.0) | 183(3.5) | 5(0.6) | 16.9 | 542(4.8) | 507(6.8) | 147(5.3) | 1(0.3) | 12.4 | 0.002 |
| Missing | 4335(23.8) | 799(19.3) | 909(17.2) | 141(17.1) | 17.0 | 2476(21.9) | 1339(18.0) | 431(15.7) | 42(13.3) | 11.0 | <0.001 |
| **Death or not** |  |  |  |  |  |  |  |  |  |  |  |
| Not | 17589(96.7) | 4079(98.3) | 4801(91.0) | 613(74.5) | 20.0 | 11247(99.3) | 7415(99.6) | 2676(97.3) | 276(87.6) | 13.7 | <0.001 |
| Yes | 603(3.3) | 69(1.7) | 477(9.0) | 210(25.5) | 50.6 | 79(0.7) | 31(0.4) | 73(2.7) | 39(12.4) | 50.5 | 0.978 |
| **Date of onset** |  |  |  |  |  |  |  |  |  |  |  |
| Before Dec 31, 2019 | 31(0.2) | 6(0.1) | 22(0.4) | 12(1.5) | 47.9 | 17(0.2) | 45(0.6) | 19(0.7) | 7(2.2) | 29.6 | 0.018 |
| Jan 1–10, 2020 | 153(0.8) | 37(0.9) | 146(2.8) | 71(8.6) | 53.3 | 112(1.0) | 145(1.9) | 78(2.8) | 17(5.4) | 27.0 | <0.001 |
| Jan 11–20, 2020 | 1288(7.1) | 141(3.4) | 792(15.0) | 186(22.6) | 40.6 | 874(7.7) | 640(8.6) | 382(13.9) | 65(20.6) | 22.8 | <0.001 |
| Jan 21–31, 2020 | 8997(49.5) | 986(23.8) | 2781(52.7) | 386(46.9) | 24.1 | 4347(38.4) | 2105(28.3) | 1131(41.1) | 121(38.4) | 16.3 | <0.001 |
| Feb 1–10, 2020 | 6140(33.8) | 1985(47.9) | 1260(23.9) | 148(18.0) | 14.8 | 4682(41.3) | 2877(38.6) | 926(33.7) | 79(25.1) | 11.7 | <0.001 |
| Feb 11–20, 2020 | 1391(7.6) | 940(22.7) | 264(5.0) | 18(2.2) | 10.8 | 1294(11.4) | 1634(21.9) | 213(7.7) | 26(8.3) | 7.6 | <0.001 |
| Feb 21–24, 2020 | 191(1.0) | 53(1.3) | 11(0.2) | 0(0.0) | 4.3 | 0(0.0) | 0(0.0) | 0(0.0) | 0(0.0) | —— | —— |
| Missing | 1(0.0) | —— | 2(0.0) | 2(0.2) | 80.0 | 43(0.4) | 18(0.2) | 9(0.3) | —— | 12.9 | —— |
| **Days from onset to diagnosis, median (IQR)** | 8(4–12) | 9(5–15) | 11(7–15) | 11.5(7–16) |  | 11(6–17) | 9(3–17) | 15(9–21) | 15.5(9–23) | —— | —— |
| **District of residence** |  |  |  |  |  |  |  |  |  |  |  |
| City centre | 14429(79.3) | 3285(79.2) | 4447(84.3) | 685(83.2) | 22.5 | 8550(75.5) | 5988(80.4) | 2163(78.7) | 219(69.5) | 14.1 | <0.001 |
| Suburb | 3625(20.0) | 821(19.8) | 757(14.3) | 116(14.1) | 16.4 | 2702(23.9) | 1395(18.7) | 559(20.3) | 92(29.2) | 13.7 | <0.001 |
| Outside Wuhan | 138(0.8) | 42(1.0) | 69(1.3) | 17(2.1) | 32.3 | 74(0.7) | 63(0.8) | 27(1.0) | 4(1.3) | 18.5 | 0.002 |
| Missing | —— | —— | 5(0.1) | 5(0.6) | 100.0 | —— | —— | —— | —— | —— |  |
| **Level of hospital** |  |  |  |  |  |  |  |  |  |  |  |
| Tertiary hospital | 13242(72.8) | 2720(65.6) | 4033(76.4) | 636(77.4) | 22.6 | 9743(86.0) | 5948(79.9) | 2528(92.0) | 267(84.8) | 15.0 | <0.001 |
| Primary/ secondary hospital | 4951(27.2) | 1428(34.4) | 1245(23.6) | 186(22.6) | 18.3 | 1583(14.0) | 1498(20.1) | 221(8.0) | 48(15.2) | 8.0 | <0.001 |

***Note.***^a^ IQR=interquartile range.

**Table S4.** Risk Factors for the Severity of COVID-19 Evaluated by Univariable Logistic-regression Analysis*

| **Variable** | **Confirmed Cases** | | **Clinically Diagnosed Cases** | |
| --- | --- | --- | --- | --- |
|  | **OR(95%CI)** | ***P*** | **OR(95%CI)** | ***P*** |
| Age, years |  |  |  |  |
| <60 | 1(reference) |  | 1(reference) |  |
| ≥60 | 2.45(2.32-2.6) | <0.001 | 2.87(2.65-3.10) | <0.001 |
| Sex |  |  |  |  |
| Female | 1(reference) |  | 1(reference) |  |
| Male | 1.12(1.06-1.18) | <0.001 | 1.16(1.07-1.25) | <0.001 |
| Occupation |  |  |  |  |
| Others | 1(reference) |  | 1(reference) |  |
| Housework or unemployed | 2.27(2.06-2.51) | <0.001 | 1.67(1.47-1.89) | <0.001 |
| Retirees | 2.69(2.46-2.94) | <0.001 | 2.63(2.34-2.96) | <0.001 |
| Healthcare worker | 1.39(1.17-1.66) | <0.001 | 1.44(1.18-1.75) | <0.001 |
| Date of onset |  |  |  |  |
| Before Dec 31, 2019 | 1(reference) |  | 1(reference) |  |
| Jan 1–10, 2020 | 1.24(0.75-2.06) | 0.398 | 0.88(0.53-1.48) | 0.631 |
| Jan 11–20, 2020 | 0.75(0.46-1.2) | 0.222 | 0.70(0.44-1.13) | 0.143 |
| Jan 21–31, 2020 | 0.35(0.22-0.55) | <0.001 | 0.46(0.29-0.73) | 0.001 |
| Feb 1–10, 2020 | 0.19(0.12-0.3) | <0.001 | 0.32(0.2-0.5) | <0.001 |
| Feb 11–24, 2020 | 0.12(0.08-0.20) | <0.001 | 0.20(0.12-0.31) | <0.001 |
| District of residence |  |  |  |  |
| Suburb | 1(reference) |  | 1(reference) |  |
| City centre | 1.48(1.36-1.60) | <0.001 | 1.03(0.94-1.13) | 0.52 |
| Level of hospital |  |  |  |  |
| Secondary and lower level | 1(reference) |  | 1(reference) |  |
| Tertiary hospital | 1.3(1.22-1.39) | <0.001 | 2.04(1.79-2.33) | <0.001 |

***Note.*** *The COVID-19 Cases with “Mild” and “Moderate” severity are defined as non-severe, and the COVID-19 Cases with “Severe” and “Critical” severity are defined as severe.

**Table S5.** Risk Factors for Death from COVID-19 Evaluated by Univariable Logistic-regression Analysis

| **Variable** | **Confirmed cases** | | **Clinically diagnosed cases** | |
| --- | --- | --- | --- | --- |
|  | **OR(95%CI)** | ***P*** | **OR(95%CI)** | ***P*** |
| **Age, years** |  |  |  |  |
| <60 | 1(reference) |  | 1(reference) |  |
| ≥60 | 6.67(5.81-7.65) | <0.001 | 7.34(5.36-10.06) | <0.001 |
| **Sex** |  |  |  |  |
| Female | 1(reference) |  | 1(reference) |  |
| Male | 1.94(1.75-2.16) | <0.001 | 2.6(1.99-3.38) | <0.001 |
| **Occupation** |  |  |  |  |
| Others | 1(reference) |  | 1(reference) |  |
| Housework or unemployed | 2.21(1.79-2.73) | <0.001 | 2.01(1.26-3.19) | 0.003 |
| Retirees | 3.65(3.02-4.4) | <0.001 | 3.25(2.11-5.01) | <0.001 |
| Healthcare worker | 0.46(0.25-0.83) | 0.01 | 0.31(0.07-1.31) | 0.112 |
| **Case severity** |  |  |  |  |
| Mild | 1(reference) |  | 1(reference) |  |
| Moderate | 0.49(0.38-0.63) | <0.001 | 0.6(0.39-0.9) | 0.015 |
| Severe | 2.9(2.56-3.28) | <0.001 | 3.88(2.82-5.35) | <0.001 |
| Critical | 9.99(8.38-11.92) | <0.001 | 20.12(13.46-30.06) | <0.001 |
| **Date of onset** |  |  |  |  |
| Before Dec 31, 2019 | 1(reference) |  | 1(reference) |  |
| Jan 1–10, 2020 | 1.05(0.67-1.66) | 0.829 | 0.52(0.16-1.74) | 0.289 |
| Jan 11–20, 2020 | 0.57(0.37-0.87) | 0.009 | 0.34(0.12-0.98) | 0.046 |
| Jan 21–31, 2020 | 0.22(0.15-0.34) | <0.001 | 0.27(0.1-0.76) | 0.013 |
| Feb 1–10, 2020 | 0.08(0.05-0.13) | <0.001 | 0.22(0.08-0.61) | 0.004 |
| Feb 11–24, 2020 | 0.03(0.02-0.05) | <0.001 | 0.22(0.08-0.65) | 0.006 |
| **District of residence** |  |  |  |  |
| Suburb | 1(reference) |  | 1(reference) |  |
| City centre | 1.26(1.09-1.46) | 0.002 | 0.81(0.6-1.1) | 0.176 |
| **Level of hospital** |  |  |  |  |
| Secondary and lower level | 1(reference) |  | 1(reference) |  |
| Tertiary hospital | 1.56(1.37-1.77) | <0.001 | 0.71(0.52-0.96) | 0.028 |


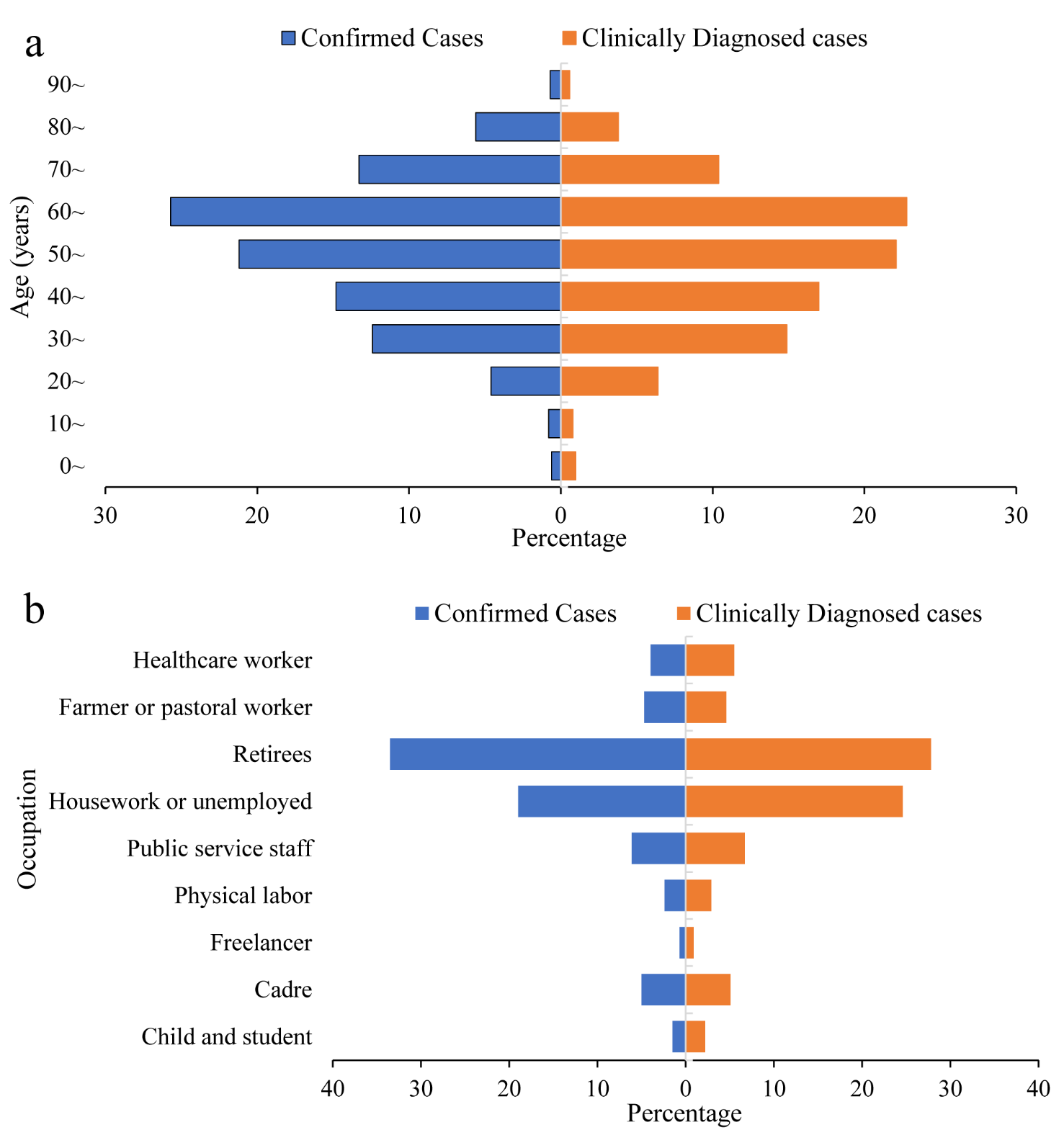


**Fig. S1 The age distribution (a) and occupation distribution (b) of confirmed cases and clinically diagnosed cases**


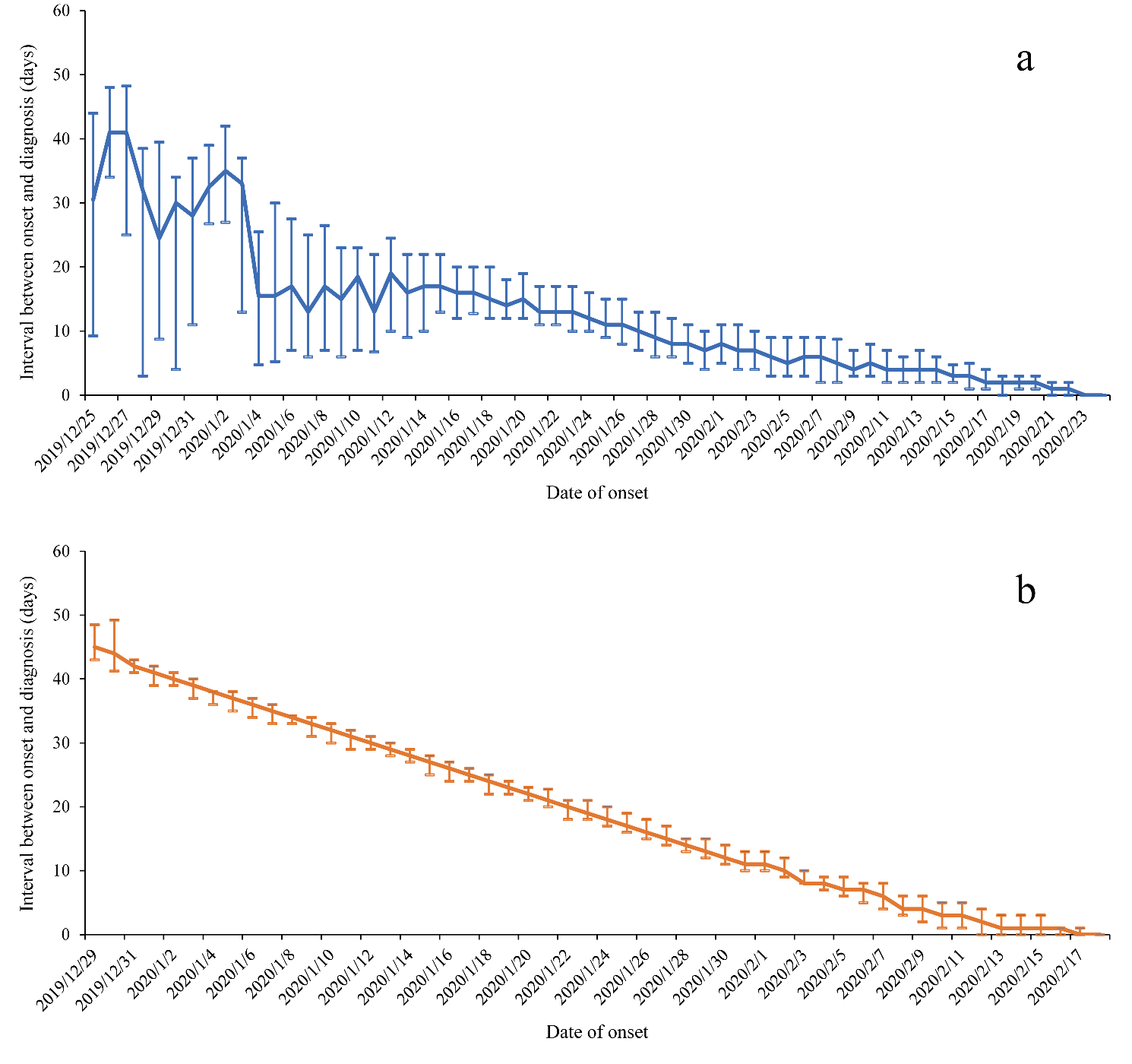


**Fig. S2** Time trend of interval between onset and diagnosis. (a) confirmed cases, (b) clinically diagnosed cases


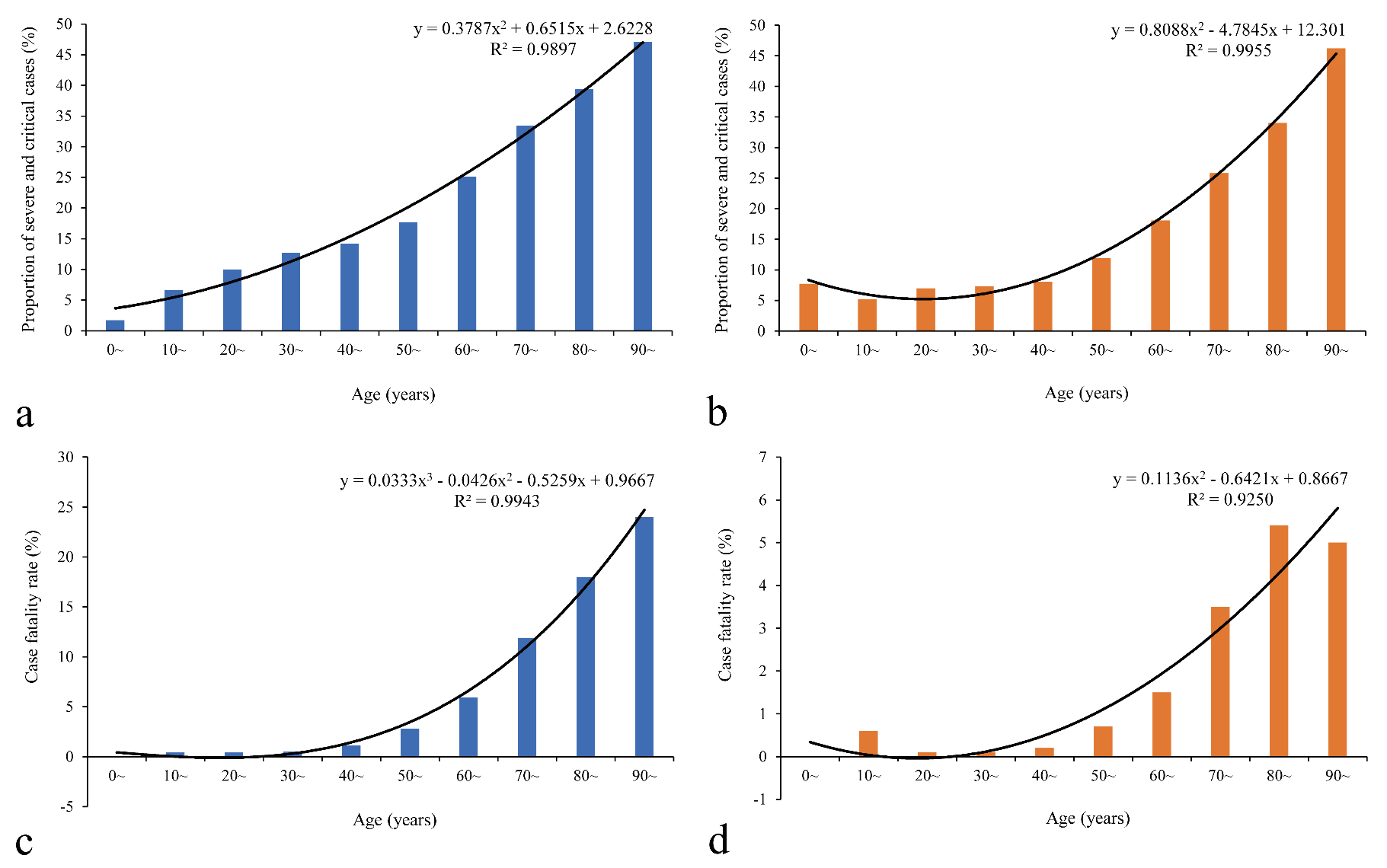


**Fig. S3** The relationship between age and disease severity / case fatality rate in COVID-19 patients. (a) the relationship between age and disease severity in confirmed cases, (b) the relationship between age and disease severity in clinically diagnosed cases, (c) the relationship between age and case fatality rate in confirmed cases, (b) the relationship between age and case fatality rate in clinically diagnosed case.


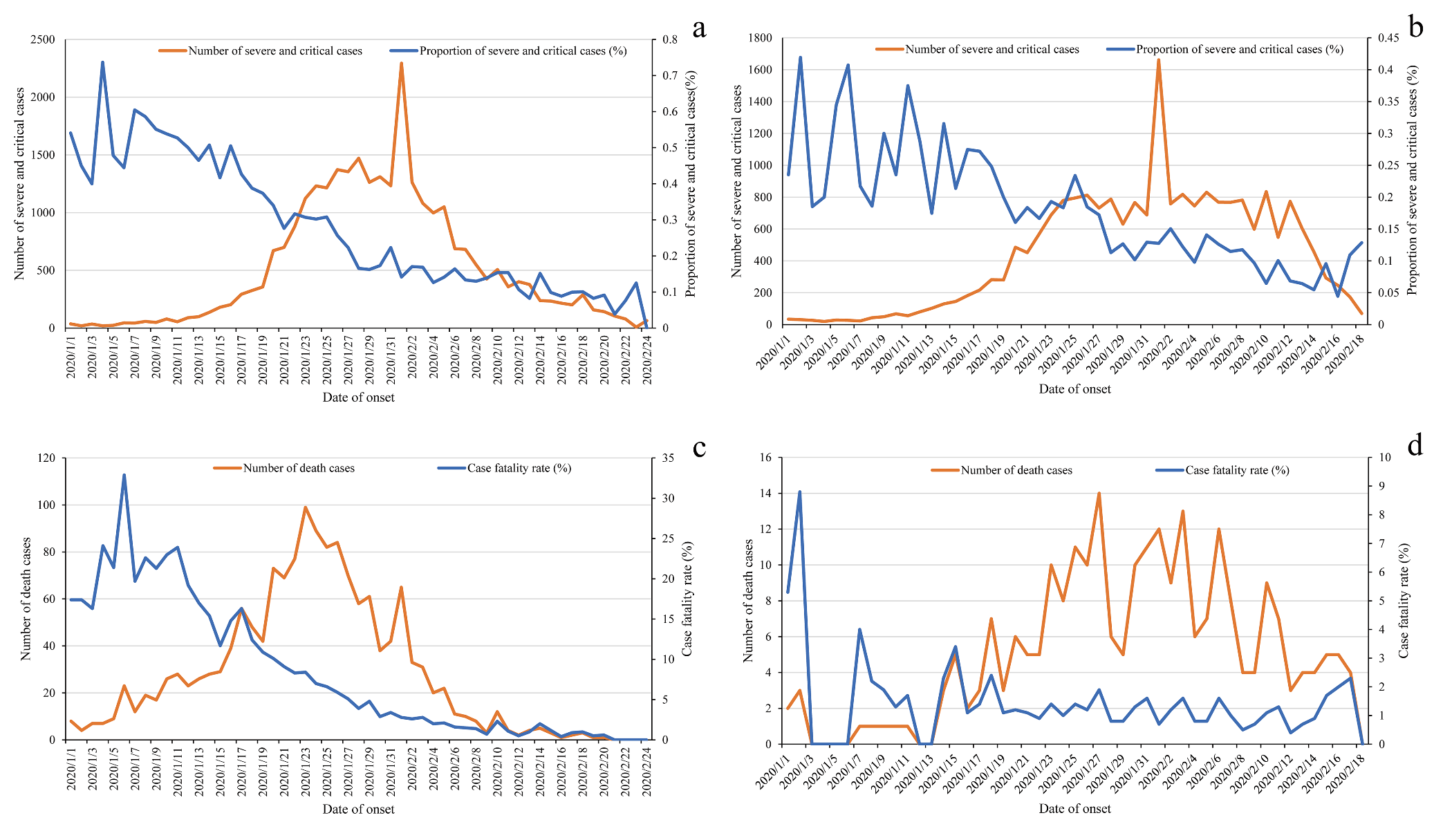


**Fig. S4** Trends in severity and death over time. (a) trends in severity of confirmed cases, (b) trends in severity of clinically diagnosed cases, (c) trends in case fatality rate of confirmed cases, (d) trends in case fatality rate of clinically diagnosed cases.


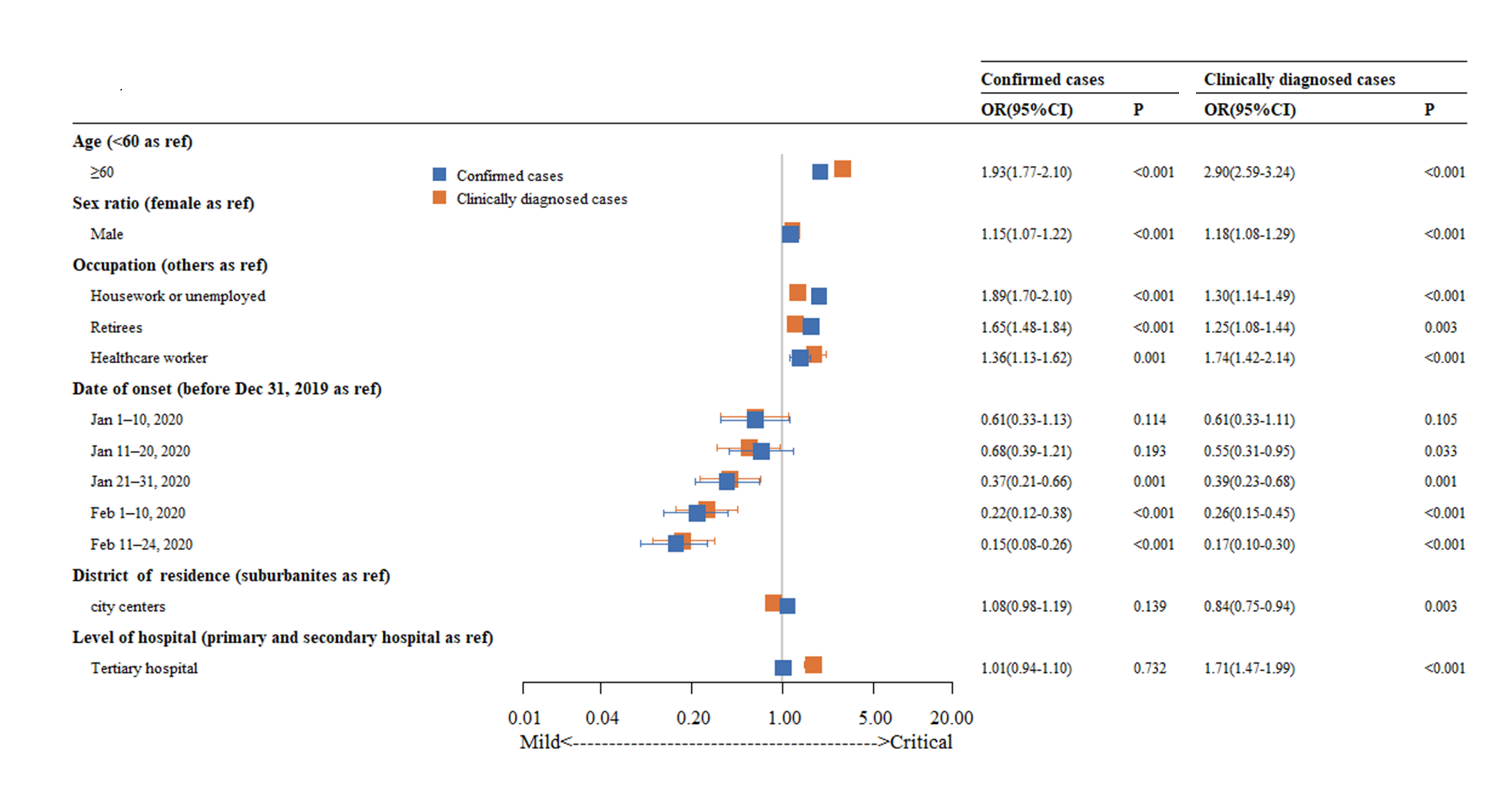


**Fig. S5** Risk factors for severity in COVID-19 patients from multivariable logistic-regression analysis. The 95% confidence intervals of the odds ratios and p-value of logistic-regression analysis are shown.


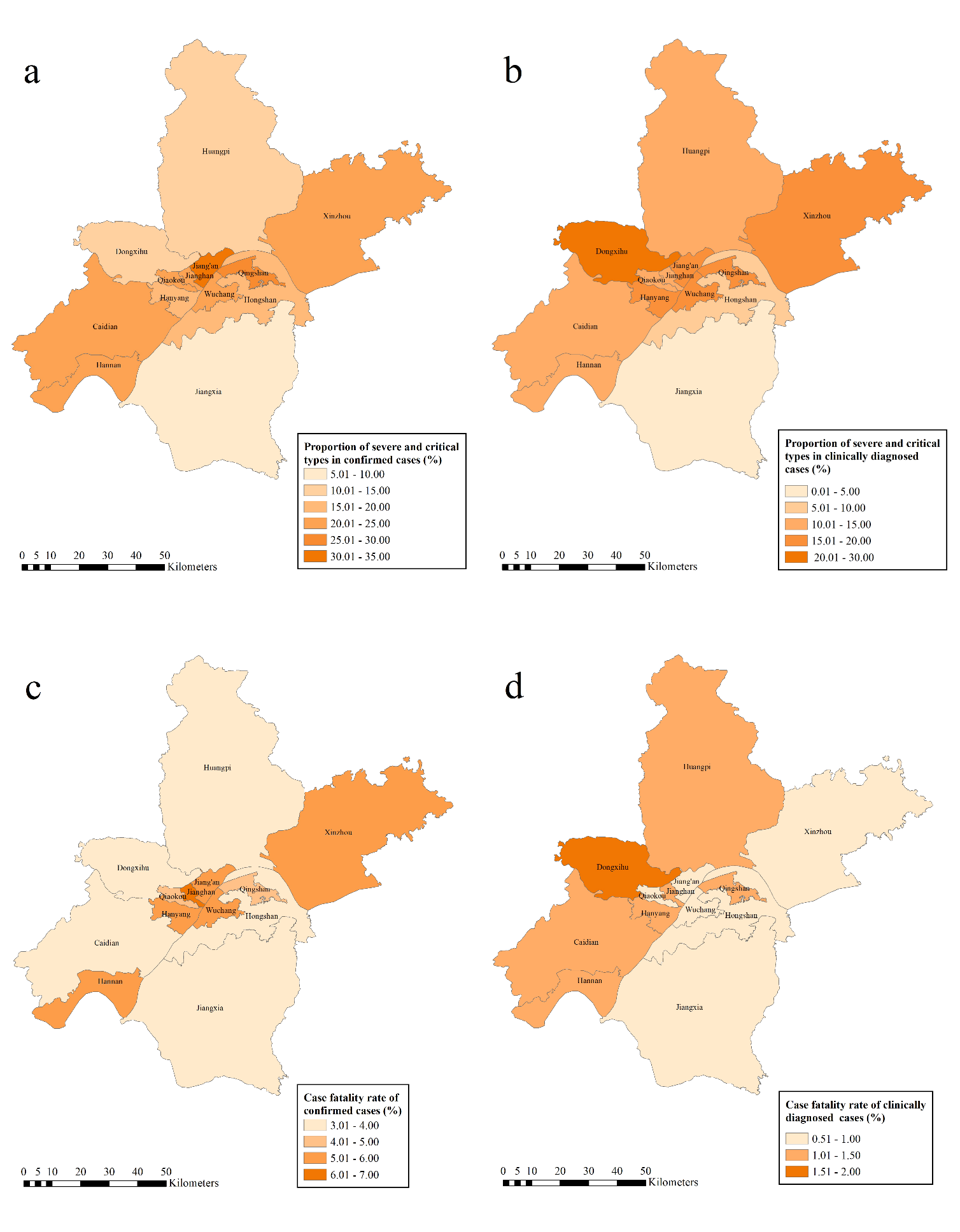


**Fig. S6** The geographical disparity of disease severity and case fatality rates. (a) proportion of severe and critical types in confirmed cases, (b) proportion of severe and critical types in clinically diagnosed cases, (c) case fatality rate of confirmed cases, (d) case fatality rate of clinically diagnosed cases


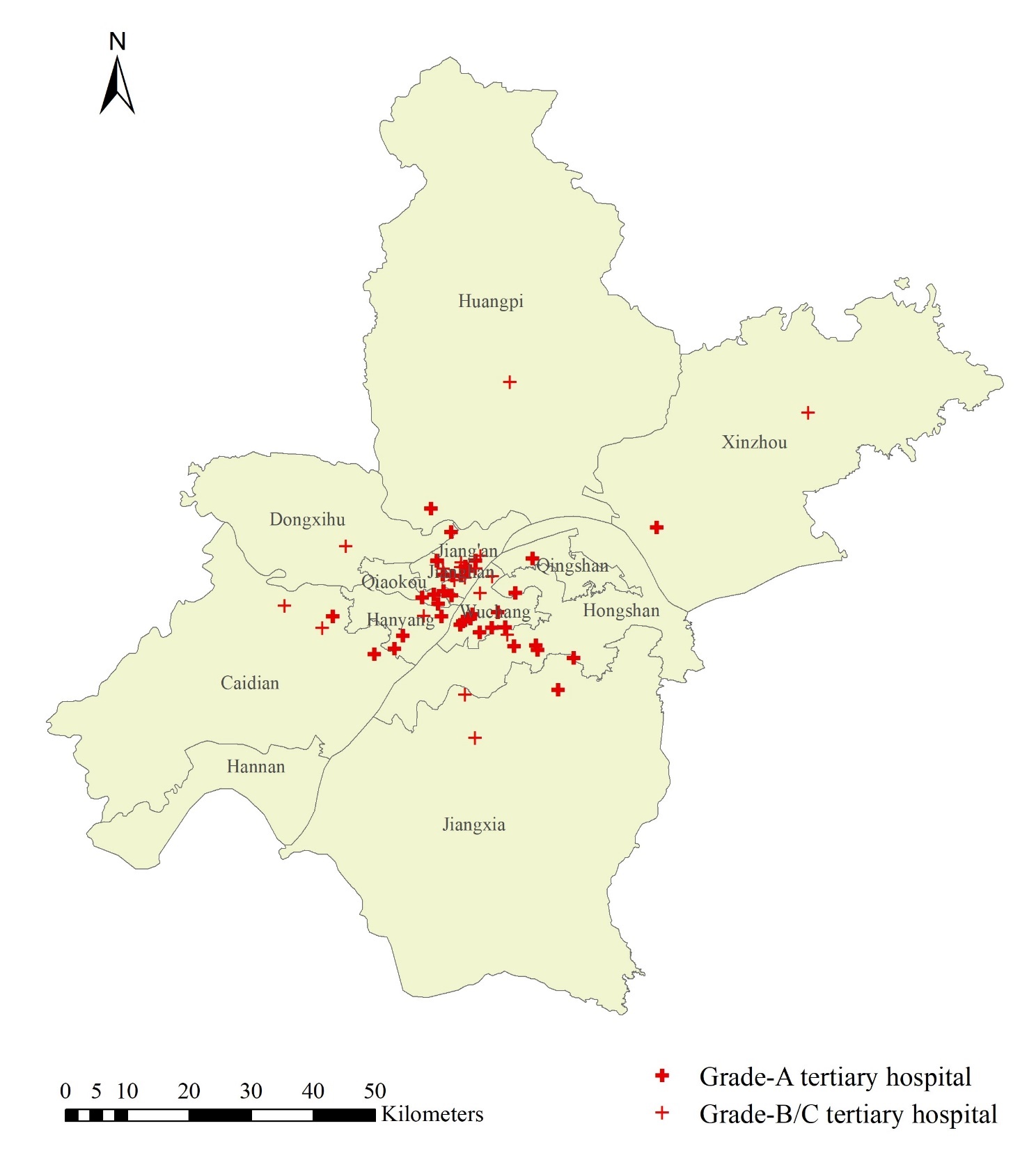


**Fig. S7** The Distribution map of tertiary hospitals in Wuhan, China
